# Occupational health risks of barbers and coiffeurs in Izmir

**DOI:** 10.4103/0019-5278.55128

**Published:** 2009-08

**Authors:** Aliye Mandiracioglu, Sukran Kose, Ayhan Gozaydin, Melda Turken, Lutfiye Kuzucu

**Affiliations:** 1 Department of Public Health of Medical Faculty, Ege University, Tepecik Training and Research Hospital, Izmir; 2 Department of Infectious Diseases and Clinic Microbiology, Tepecik Training and Research Hospital, Izmir

**Keywords:** Hairdresser, barber, chemical safety, occupational health, prevention

## Abstract

The objective of this study was to examine self-reported occupational health risks and health complaints of barbers and hairdressers. A total of 1284 individuals from 300 workplaces in Izmir participated in this study. The workers completed the questionnaires during their training in occupational health. Self-reported symptoms were allergy: 35% and musculoskeletal symptoms: 32%. The frequency of allergy complaints was found to be significantly higher in older individuals and in women. Allergic complaints were more frequent in i) those having history of allergy, ii) in the group where the use of protective clothing and gloves was lower, iii) in smokers and in those who found ventilation in the workplace to be inadequate. Only 41.2% reported that they used gloves and 15.2% reported the use of protective clothing within the last month. It appears that poor occupational factors in barbers' salons and exposure to hairdressing chemicals bring about health problems of the hairdressers.

## INTRODUCTION

Barbers and hairdressers are subjected to various occupational health risks. Problems such as poor posture, mechanical load on the joints, prolonged standing, longer working hours, missed meals, not taking breaks during working, as well as being subjected to physical factors such as noise and higher temperatures are important occupational health risks for these people.[1,2]

Cosmetic products such as shampoos, creams, hair dyes, sprays, and hair conditioners that contain hundreds of chemicals, are being used in barbers' and hairdressers' salons. Some of the chemicals which barbers are exposed to are: *p*-benzenediamine, *o*-benzenediamine, *p*-phenylenediamine, *p*-toluenediamine, toluene-2,5-diamine, *p*-aminophenol, *m*-aminophenol, parabens, formaldehyde, methylisothiazolinones, ethanol, isopropanol, ammonia, phenols, alcohols, persulphates monoethanolamine, glyceryl monothioglycolate, ammonium thioglycolate, ammonium chloride or ammonium phosphate, hydrogen peroxide, formaldehyde, carbon dioxide, and carbon monoxide.[3–11]

Chemicals with their allergenic and irritant effects frequently cause health problems such as respiratory tract reactions, asthma, dermatitis, rhinitis, and ocular diseases in barbers[3,5,12–20] It is also known that chemicals affect hairdressers' reproductive health and contribute to indoor air pollution.[2,21,22]

Ronda *et al*. in their indoor air measurements carried out in hairdressing salons, have detected higher levels of benzene, dichloromethane, and ethylbenzene, all known carcinogens. They also detected hexane, benzene, methoxypropanol, and toluene that are thought to affect reproductive health, as well as potential allergens such as diethylphthalate and limonene. It has been emphasized that the most important problem in these salons is the consequences that may arise from concomitant exposure to multiple chemicals.[6] It has been reported that the inner atmosphere of the salons are not only chemically unsafe, but also environmentally unsafe in terms of temperature, humidity, lighting, and ventilation.[6,7,23] Workers' electrical and electromagnetic exposures were found to be high in this study.[23]

In addition, workers in beauty parlors and hairdressers' and barbers' salons are likely to have contact with blood through processes such as cutting, manicure, pedicure, and skin care. A number of studies cautioned that if necessary emphasis was not given by these personnel to their personal hygiene, decontamination, disinfection, sterilization of working equipment, disposal of waste and sanitary conditions of the working environment, they may be under increased risk of spreading certain diseases, not only to themselves, but also to their customers.[24–28] The aim of this study was to examine self-reported occupational health risks and complaints of barbers and hairdressers.

## MATERIALS AND METHODS

This cross-sectional study was carried out in Izmir province on the Mediterranean coast in 2006. Male and female hairdressers and beauty parlors located in metropolitan counties in the heavily populated central part of the city were included within the scope of this study. Among these, 1284 individuals (workplace owners, shop masters, assistants, and apprentices) from 300 workplaces registered to the Barbers and Hairdresser Trade Corporation of Izmir participated.

Individuals were asked to complete survey forms using the self-report method. Questions asked aimed to identify gender, age, occupational position, and length of time spent in this occupation as well as to collect information concerning workplace conditions (area of the workplace, number of employees, ventilation characteristics), occupational practices, substances used (hair dye, spray etc.), frequency of exposure to chemicals, use of gloves, presence of any painful disorder, presence of allergic symptoms, personal and familial allergy history. Questionnaires were handed out during a training program and were completed by the participants.

The data of the study were evaluated with SPSS computer program, version 13.0.

## RESULTS

The mean age of the participants was calculated as 29.44 ± 10.29 years (range: 12–77 years) and 9.3% of them were younger than 18 years old. Over half (59.9%) were males and 40.8% had elementary or lower education levels. The mean number of years of experience in this occupation was 12.88 ± 9.28 years (range: 1–55 years).

Of the participants, 56.1% were salon and parlor owners and out of the workplaces, 15.1% had first class, 53.3% second, and the remaining third class barber status. The mean number of customers was 19.20 ± 14.07 (range: 1–100); 12.0% of the workers stated that they performed manicures/pedicures and other beauty processes, 59.5% performed hair processes, and the rest performed all types of work.

The average area of the workplace was 54.77 ± 55.33 m^2^ (range: 8–450 m^2^). It was reported that all the workplaces were window and/or door-ventilated. Air-conditioning was used for heating and cooling in the majority of the workplaces (70%). Most (82.7%) of the workers stated that they found the ventilation of their workplaces to be adequate. Over half (54.3%) of the workers were smokers and all of them, except for five workers, stated that smoking was allowed in their workplaces.

The question related to chemical usage status was answered only by 391 individuals [Table 1]. The use of hair colorings and hair spray were most common, but daily mean usage was lower. On the other hand, the percentage of employees who used shampoos was not high although mean usage was higher.

**Table 1:** Characteristics of the chemicals used by study participants

At least one usage daily	Employee %	Mean ± SD	min–max
Hair dye	75.9	4.14± 3.70	1–30
Spray	84.1	6.67±5.54	1–30
Hair gel or cream	83.0	8.17±8.028	1–35
Powder	82.0	6.21±6.63	0–40
Acetone	50.2	5.13±4.997	1–50
Hair tone lightener	46.2	3.82±7.501	0–100
Shampoo	44.5	8.99±9.722	1–80
Perm material	43.1	2.66±2.658	0–15

Only 41.2% of these workers reported that they used gloves and 15.2% had used protective clothing within the last month. It was determined that 4.4% practised dry air sterilization and 3.4% allowed their customers to use their own equipment. The vast majority (80.9%) reported that they used an ultraviolet sterilization device whereas others preferred to wash their equipment with soapy water (9.2%), wipe it with alcohol (41.7%), or to immerse it in a disinfectant chemical (12.7%). Only one-third of these employees washed their hands before and after each process.

When the respondents were asked to identify any discomfort in the past 12 months, 35.0% stated that they had at least one allergic complaint. Nearly 32% all in the groups complained of musculoskeletal discomfort; among these, back pain (27.4%) and lumbago (25.5%) were the most frequent. The participants reported some other health problems: headaches (5.8%), varicosis (15.5%), and fungal infection (1.1%).

Approximately half of those having an allergic history could specify the causes of the allergy—the vast majority consisted of cosmetics and dust allergies. The prevalence of allergic symptoms is seen in Table 2. The most frequent symptom was watery eyes. In older individuals (chi-square: 18.462, *P* < 0.001) and in women (chi-square: 14.084, *P* < 0.001), the frequency of allergic complaints was found to be significantly higher. Allergic complaints were more frequent in i) those having a history of allergy (chi-square: 75.02, *P* < 0.03), ii) in the group where the use of protective clothing and gloves was lower (chi-square: 8.06, *P* < 0.001), iii) in smokers (chi-square: 10.693, *P* < 0.005), iv) and in those who found the ventilation in the workplace to be inadequate (chi-square: 26.27, *P* < 0.0001). It was not found to be related to the number of years of service. Allergic complaints were infrequent only in those performing manicures, pedicures, and beauty care (chi-square: 6.578, *P* < 0.037). The mean number of customers and the mean area of these workplaces were not found to be related to the frequency of allergic complaints.

**Table 2:** Frequency of allergic complaints of study participants within the last month

Allergic complaints	Frequency (%)
Flushing on skin	15.6
Cleavage on skin	8.7
Dryness and squama on skin	12.8
Rash formation on skin	4.2
Pruritus on skin	10.9
Dyspnea	18.7
Hay fever	5.8
Coughing longer than two weeks	4.8
Watery eyes	35.0

## DISCUSSION

This study examined the occupational health risks of individuals working in barbers' and hairdressers' salons in Izmir. The daily use of cosmetics containing chemicals was found to be considerably high. On the other hand, it was found that workers did not use measures that would reduce their exposure to these chemicals. General window ventilation was used for the ventilation of these workplaces and local ventilation does not exist in any of the salons. Studies have already proposed that some complaints may increase with inappropriate indoor ventilation.[2] The rate of exposure to chemicals could be reduced by improving ventilation. It has been reported that although individual chemical exposure rates are low, concurrent exposure to multiple chemicals was not taken into consideration. Exposure rates have been shown to increase in smaller and busy workplaces. It is recommended that both the sections where dyes are prepared, besides the whole workplace, should be ventilated.[6,7,10]

Health risks related to exposure to environmental tobacco smoke are well recognized. The workplace is the primary source of exposure to environmental tobacco smoke among nonsmoker adults.[20] The authors of this study found that the prevalence of the smoking habit was higher in these workers than in those of other sectors. It has been reported that 34.6% of Turkish adults are smokers.[30] The law prohibiting smoking in closed areas in Turkey was put into effect on 19 May, 2008, so it was not yet in effect when this study was carried out. Smoking in the workplace and in public places was common until the introduction of a comprehensive antismoking law in May 2008.

It has been identified that barbers and hairdressers do not pay sufficient attention to the use of protective clothing and gloves, which are highly important for skin protection and exposure prevention. Our study, like others,[6,7,16,19,31] found that gloves are often not used during the washing of customers' hair.

The overall low use of gloves demonstrates that these workers are poorly protected from biological factors as well as chemicals. In addition, this study also found that sanitation and sterilization of equipment used in workplace, hand-washing, and the wearing of protective clothing were not satisfactory. Earlier studies have already shown inappropriate protection against blood-borne diseases in barbers and hairdressers.[24–28]

It was determined that there were more rhinitis, ocular symptoms, and respiratory tract complaints in barbers and hairdressers than in the general population.[32,35] In this study, 35% of the participants had at least one allergic complaint. Some studies have reported a lower frequency of symptoms than expected, which could be attributed to the fact that those with impaired health quit their jobs.[19] In agreement with other studies,[32,36] our study did not find any relationship between the number of years in service and the frequency of symptoms, which may be interpreted as a “healthy worker effect.” In Turkey, people begin this occupation at early ages and these salons have a high staff turnover. It has been reported that the risk of having to quit the job due to an allergic disease was > 20% among hairdressers.[37]

In this study, an increase in the frequency of complaints was found in those having an atopic history. Leino *et al*. found that the risk of emergence of occupational respiratory tract and cutaneous complaints was three times higher in atopic individuals.[38]

However, like other studies,[35] our study found fewer symptoms in smokers.

Less than half (32.1%) of the individuals interviewed in our study had muscle-skeleton complaints, a finding that was consistent with the findings of other studies.[1,7] Biomechanical, organizational, and psychosocial factors have been proposed to play a role in these complaints due to .the load and coercion on the shoulders, wrists, and other joints, which could be reduced by ergonomic hand devices.[1]

Although this study covered a larger sample in Izmir, the limitations of this study included the lack of clinical examinations and a dependence on self-reporting by the participants. Recall bias might be a problem in survey studies. Besides, a study design which will establish an association between reported complaints and occupational health risks, has not been formulated. No inhouse observations were preformed; the data in this study were collected during an occupational health seminar directed at this sector.

Hairdressers and barbers are mostly at an increased risk of developing cutaneous, respiratory tract, musculo-skeleton system, and blood-borne diseases, hence, occupational measures should be implemented to lower these risks.[6,7] Awareness should be built in these workers about the hazards of the chemicals they could be exposed to and atopic individuals should be warned about the risks involved in picking this occupation.[5,10,11] It has been reported that the frequency of occupational cutaneous diseases decreased in Northern Bavaria through occupational health training in Germany.[39]

The environment of the workplace should be safe: improving the ventilation, replacement of cosmetics containing hazardous chemicals with other cosmetic products, production and use of less hazardous cosmetics, and imposition of legal sanctions in this regard are advised.[10]

It has been suggested that success can be achieved in the use of safer products by regulating the use of cosmetics which contain chemical substances.[5]

There are occupational apprentice centers in Turkey for the occupational training of hairdressers and barbers. Younger people who graduate from elementary school (i.e., who have completed an eight-year basic education] and who are younger than 19 years of age, can work in a workplace with social security while taking courses in these places. During this education, they receive training concerning occupational health risks. Hairdressers and barbers take their licenses from the local municipality according to the “Regulation related to Permission to Open and Run a Business for Sanitary Enterprises”. However, inspections and legal arrangements are not sufficient; the Turkish Barbers and Hairdressers Federation should disseminate guidelines related to the work of barbers and hairdressers.

## References

[1] Mussi G, Gouveia N (2008). Prevalence of work-related musculoskeletal disorders in Brazilian hairdressers. Occup Med (Lond).

[2] Hollund BE, Moen BE, Lygre SH, Florvaag E, Omenaas E (2001). Prevalence of airway symptoms among hairdressers in Bergen, Norway. Occup Environ Med.

[3] Hollund BE, Moen BE, Egeland GE, Florvaag E (2003). Prevalence of airway symptoms and total serum immunoglobulin E among hairdressers in Bergen: A four-year prospective study. J Occup Environ Med.

[4] Søsted H, Rastogi SC, Andersen KE, Johansen JD, Menné T (2004). Hair dye contact allergy: Quantitative exposure assessment of selected products and clinical cases. Contact Dermatitis.

[5] Lee A, Nixon R (2001). Occupational skin disease in hairdressers. Australasian Journal of Dermatology.

[6] Ronda E, Hollund BE, Bente E (2008). Airborne exposure to chemical substances in hairdresser salons. Environ Monit Assess.

[7] Leino T, Kähkönen E, Saarinen L, Henriks-Eckerman ML, Paakkulainen H (1999). Working conditions and health in hairdressing salons. Applied Occupational and Environmental Hygiene.

[8] Van der Wal JF, Hoogeveen AW, Moons AMM, Wouda P (1997). Investigation on the exposure of hairdressers to chemical agents. Environment International.

[9] Hollund BE, Moen BE (1998). Chemical Exposure in Hairdresser Salons: Effect of Local Exhaust Ventilation Ann. Hyg.

[10] Mounier-Geyssant E, Oury V, Mouchot L, Paris C, Zmirou-Navier D (2006). Exposure of hairdressing apprentices to airborne hazardous substances. Environmental Health: A Global Access Science Source.

[11] Wong RH, Chien HL, Luh DL, Lin WH, Wang YC, Cho CY (2005). Correlation Between Chemical-Safety Knowledge and Personal Attitudes Among Taiwanese Hairdressing Students. Am. J. Ind. Med.

[12] Palmer KT, Freegard J (1996). Compliance with the Control of Substances Hazardous to Health Regulations (COSHH) 1988 and health and safety awareness in hairdressing establishments. Occup Med (Lond).

[13] Anveden I, Lidén C, Alderling M, Meding B (2006). Self-reported skin exposure: Validation of questions by observation. Contact Dermatitis.

[14] Khumalo NP, Jessop S, Ehrlich R (2006). Prevalence of cutaneous adverse effects of hairdressing: A systematic review. Arch Dermatol.

[15] Schröder CM, Merk HF, Frank J (2006). Barber's hair sinus in a female hairdresser: uncommon manifestation of an occupational dermatosis. J Eur Acad Dermatol Venereol.

[16] Lind ML, Boman A, Sollenberg J, Johnsson S, Hagelthorn G, Meding B (2005). Occupational dermal exposure to permanent hair dyes among hairdressers. Ann Occup Hyg.

[17] Holm JO, Veierød MB (1994). An epidemiological study of hand eczema.V. Prevalence among hairdresser trainees, compared with a general population of hairdressers. Acta Derm Venereol Suppl (Stockh).

[18] Lind ML, Albin M, Brisman J, Kronholm Diab K, Lillienberg L, Mikoczy Z (2007). Incidence of hand eczema in female Swedish hairdressers. Occup Environ Med.

[19] Ling TC, Coulson IH (2002). What do trainee hairdressers know about hand dermatitis?. Contact Dermatitis.

[20] Brisman J, Albin M, Rylander L, Mikoczy Z, Lillienberg L, Höglund AD (2003). The incidence of respiratory symptoms in female Swedish hairdressers. Am J Ind Med.

[21] Kersemaekers WM, Roeleveld N, Zielhuis GA (1995). Reproductive disorders due to chemical exposure among hairdressers. Scandinavian Journal of Work, Environment and Health.

[22] Reijula K, Sundman-Diger C (2004). Assessment of indoor air problems at work with a questionnaire. Occup Environ Med.

[23] Evci ED, Bilgin MD, Akgör S, Zencirci SG, Ergiń F, Beşer E (2007). Measurement of selected indoor physical environmental factors in hairdresser salons in a Turkish City. Environ Monit Assess.

[24] Murtagh MJ, Hepworth J (2004). Hepatitis C in the workplace; a survey of occupational health and safety knowledge and practice in the beauty industry. Aust. N.Z.J. Public Health.

[25] Tumminelli F, Marcelin P, Rizzo S, Barbera S, Corvino G, Furia S (1995). Shaving as potential source of hepatitis C virus infection. The Lancet.

[26] Moore JE, Cherie B, Miller BC (2007). Skin, hair and other infections associated with visits to barber's shops and hairdressing saloons. AJIC.

[27] Abdul Khaliq A, Smego RA (2005). Barber shaving and blood-borne disease transmission in developing countries. SAMJ.

[28] Baakrim MZ, Laraqui S, Laraqui O, El Kabouss Y, Verger C, Caubet (2002). Infectious risks associated with blood exposure for traditional barbers and their customers in Morocco. Sante.

[29] World Health Organization (1990). It can be done. A smoke-free Europe. Report of the first European Conference on TobaccoPolicy. Madrid, 7-11 November 1988. WHO Reg Publ Eur Ser.

[30] WHO Report on the global tobacco epidemic, 2008 The MPOWER package (2008).

[31] Uter W, Pfahlberg A, Gefeller O, Schwanitz HJ (1995). Occupational dermatitis in hairdressing apprentices. Early-onset irritant skin damage. Curr Probl Dermatol.

[32] Akpinar Elci M, Cimrin AH, Elci OC (2002). Prevalence and Risk Factors of Occupational Asthma Among Hairdressers in Turkey. JOEM.

[33] Leino T, Tammilehto L, Luukkonen R, Nordman H (1997). Self reported respiratory symptoms and diseases among hairdressers. Occup Environ Med.

[34] Slater T, Bradshaw L, Fishwick D, Cheng S, Kimbell-Dunn M, Erkinjuntti-Pekkanen R (2000). Occupational respiratory symptoms in New Zealand hairdressers. Occup Med.

[35] Lillienberg, Zoli Mikoczy, Jörn Nielsen, Lars Rylander, Kjell Torén, Birgitta Marie, Louise Lind (2007). Incidence of hand eczema in female Swedish hairdressers. Occup. Environ. Med.

[36] Iwatsubo Y, Matrat M, Brochard P, Ameille J, Choudat D, Conso F (2003). Healthy worker effect and changes in respiratory symptoms and lung function in hairdressing apprentices. Occup Environ Med.

[37] Leino T, Tuomi K, Paakkulainen H, Klockars M (1999). Health reasons for leaving the profession as determined among Finnish hairdressers in 1980-1995. Int Arch Occup Environ Health.

[38] Leino T, Tammilehto L, Hytönen M, Sala E, Paakkulainen H, Kanerva L (1998). Occupational skin and respiratory diseases among hairdressers. Scand J Work Environ Health.

[39] Dickel H, Kuss O, Schmidt A, Diepgen TL (2002). Impact of preventive strategies on trend of occupational skin disease in hairdressers: Population based register study. BMJ.

